# A social network analysis approach to assess COVID19-related disruption to substance use treatment and informal social interactions among people who use drugs in Scotland

**DOI:** 10.1186/s13722-024-00469-3

**Published:** 2024-05-22

**Authors:** Mark McCann, Federica Bianchi, Srebrenka Letina, Samantha Stewart, Katy McLeod, Mark Tranmer

**Affiliations:** 1 MRC/CSO Social & Public Health Sciences Unit, University of Glasgow, Glasgow, Scotland; 2 Institute of Computing, Università della Svizzera Italiana, Viganello, Switzerland; 3 Scottish Drugs Forum, Glasgow, Scotland; 4 College of Social Sciences, Institute of Health and Wellbeing, University of Glasgow, Glasgow, Scotland

**Keywords:** COVID19, Health services evaluation, Multilevel model, Social network analysis, Egonet

## Abstract

**Aims:**

To assess the extent of Coronavirus-related disruption to health and social care treatment and social interactions among people with lived or living experience of substance use in Scotland, and explore potential reasons for variations in disruption.

**Design:**

Cross sectional mixed methods interview, incorporating a social network ‘egonet interview’ approach asking about whether participants had interactions with a range of substance use, health, social care or third sector organisations, or informal social interactions.

**Setting:**

Five Alcohol and Drug Partnership Areas in Scotland.

**Participants:**

57 (42% women) participants were involved in the study, on average 42 years old.

**Measurements:**

Five-point Likert scale reporting whether interactions with a range of services and people had gotten much better, better, no different (or no change), worse, or much worse since COVID19 and lockdown. Ratings were nested within participants (Individuals provided multiple ratings) and some ratings were also nested within treatment service (services received multiple ratings). The nested structure was accounted for using cross classified ordinal logistic multilevel models.

**Findings:**

While the overall average suggested only a slight negative change in interactions (mean rating 2.93), there were substantial variations according to type of interaction, and between individuals. Reported change was more often negative for mental health services (Adjusted OR = 0.93 95% CI 0.17,0.90), and positive for pharmacies (3.03 95% CI 1.36, 5.93). The models found between-participant variation of around 10%, and negligible between-service variation of around 1% in ratings. Ratings didn’t vary by individual age or gender but there was variation between areas.

**Conclusions:**

Substance use treatment service adaptations due to COVID19 lockdown led to both positive and negative service user experiences. Social network methods provide an effective way to describe complex system-wide interaction patterns, and to measure variations at the individual, service, and area level.

**Supplementary Information:**

The online version contains supplementary material available at 10.1186/s13722-024-00469-3.

## Introduction

UK and Scottish COVID19 lockdown and restrictions led to major changes in interactions with health, social care, and community organisations, as well as affecting interactions in peoples’ social lives [1]. People with lived and living experience of substance use faced disruption to their access to formal services and informal social interactions. People who use drugs often experience syndemic ill health, with a range of poor health and social outcomes that combine and interact to worsen overall wellbeing and life expectancy [2]; and which is associated with multiple health and social support needs. The treatment gap between level of health need and level of support provided is a public health challenge [3], and assessing how lockdown restrictions affected the treatment gap and unmet need was a high priority.

Social interactions have a substantial influence on health and wellbeing, and these affects are particularly pronounced in relation to substance use and harm. Theories of social effects on substance use include social influences which encourage and facilitate drug use, and social norms, where an individual’s perceptions of others’ substance use encourages repeated or recurrent drug use over time [4]. Social and community connections can also serve as elements of recovery capital, which refers to the wider range of internal and external resources which can support recovery from substance use problems. Having social networks with more people in recovery has associated with better quality of life [5]. COVID19 and the associated change in services could impact both the heath promoting and health harming aspects of social interactions, affecting accessibility of formal services, and also reducing the size of informal social networks so that peripheral “weak ties” – sources of diversity of characteristics and information – were lost [6].

Understanding the provision of support for people who use substances, while also accounting for variation between individuals in the types of support required, is a complex issue. The widespread and varied changes introduced by COVID19 and lockdown made understanding support patterns even more challenging. Service evaluation approaches are usually designed to provide data about a single service or organisation. Qualitative approaches can explore person-centred experience of interactions with many people and organisations, but it is difficult to obtain a collective view of multiple interactions or to make inference around the relative contribution of one service compared to others. Systems science methods can provide insight into the relationship between diverse entities within a system and their interactions, while also describing the collective characteristics of the social system [7]. This project used social network methods [8] to explore system-wide effects of COVID19 on people’s experience of the substance use sector.

The ICAROS project (Impact of COVID19 and associated Response On people who use or have used Substances) developed from the need to assess how COVID19 restrictions affected this population, to facilitate mitigating action. The primary aim for the ICAROS project was to initiate a Transdisciplinary Complex Adaptive System “learning cycle” [9] by providing briefings to local public health teams and support to individuals in the areas where fieldwork took place, in order to mitigate negative consequences of COVID restrictions.

This paper reports on the analysis of the combined data for the project. The research aims were to assess the extent of disruption to service and social interactions among people who currently use or who previously used substances in Scotland and explore potential reasons for variations in disruption. Our research questions were RQ1) What individual, service, and area level factors are associated with positive and negative disruptions, and RQ2) What could explain these variations?

## Methods

### Context

What had previously been planned as a service evaluation for an Alcohol and Drug Partnership area in Scotland was reconfigured after the UK lockdown on 23rd March 2020. The reconfigured service evaluation served as a pilot and opportunity for participant and service user feedback on the study design while the academic team obtained ethical approval for a research study in more areas. We report on the full data from 1 pilot service evaluation area and 4 study areas. Interviews stopped in March 2021. The study was approved by the Ethics committee for the College of Medicine, Veterinary and Life Sciences at the University of Glasgow (Ref: 200190159).

### Design

A cross sectional mixed method egonet interview. In egonet interviewing, the participant is referred to as ‘ego’ and the individuals or organisations that ego interacts with are ‘alters’.

### Procedures

The Scottish Drugs Forum peer research team undertook fieldwork for the project; the peer research volunteers have lived or living experience of substance use and received training in interviewing and research methods.

The traditional recruitment and interview process required modification due to lockdown. The peer research team engaged their social networks to recruit study participants, and local alcohol and drug services were also asked to refer clients who met the criteria to contact the study team. Participants were asked to refer their social contacts to also take part in the study. SDF staff arranged appointment times with potential participants and e-mailed or texted study information sheets in advance of the interview. SDF staff held a three-way phone call at the appointment time, with the participant and peer researcher. To increase participant comfort and address confidentiality concerns with engaging in an uncommon interview format, the telephone interviews were not recorded and transcribed. Instead, quantitative survey answers were recorded by hand by SDF staff, and fieldwork notes taken for responses to the unstructured questions.

An SDF staff member introduced the study, assessed sobriety and capacity to consent to the study (arranging a new appointment time or cancelling where appropriate), took a record of verbal consent, and noted the participant location in case of emergency. After completing this process, the peer volunteer conducted the interview without interruption from SDF staff. After interview, SDF staff would close the interview, signposting to sources of support, or stop the interview where necessary e.g. due to participant safety concerns. After the interview, the staff member and peer interviewer debriefed and collated their notes on the verbal discussions. Participants were reimbursed with a £10 voucher for their time, and also with up to three £5 vouchers for referring other participants into the study.

### Study location

Five Alcohol and Drug Partnership areas across Scotland. There are 31 alcohol and drug partnership areas in Scotland. Each ADP has representatives from local organisations such as the health board, local authority, police and non-government organisations, and they are responsible for strategy setting and commissioning services in the local area.

### Participants

The inclusion criteria were: aged 18 +; either currently using substances, or currently engaged in treatment for substance use; and deemed to have capacity to consent to the study.

### Measures

#### Ego (participant) information

Gender, age, living arrangements, self-rated physical health and self-rated mental health, both on a five-point scale from excellent to very poor. Health variables changed after the pilot area, so we manually disaggregated physical health and mental health services in the pilot area responses (i.e. the qualitative text usually explained whether poor health was mental, physical or both). See appendix for full information on scales.

#### Alter (participant contact) information

Participants were asked a series of questions about contacts with three categories of alter: (A) Formal interactions, e.g. “do you use an addiction service, pharmacy, mental health service?”; (B) Informal interactions e.g. “are you in contact with friends, family, do hobbies, go shopping for food/to a food bank?”; and (C) Substance use interactions e.g. “Do you have a supplier for alcohol, cannabis, benzos, heroin?”.

#### Outcome variable – level of COVID19 disruption

For each type of alter reported by participants, participants were asked whether interactions had changed due to COVID19 on a five-point scale: ‘much better’ ‘better’, ‘no change or about the same’, ‘worse’, or ‘much worse’, along with open ended information on the reasons for the rating.

#### Overlapping alters

Information on the names of specific services was used to identify services that were common across participants i.e. where participants attended the same service. This ‘overlapping alter’ information was used in the analysis. Name information wasn’t available for individual pharmacies, GPs, or substance use suppliers, so the dataset is likely to under-report the true degree of overlapping alters.

#### Data analysis

Following a framework for analysing egonet data with overlapping alters [10], analyses used cross classified multilevel models. Our outcome variable was the five-point rating of change in interactions, and outcomes were nested in two different level two classifications. Ratings were nested within egos, as participants provided ratings for each of their interactions; and ratings were also nested within alters, as some alters received multiple ratings by different participants. The random effect terms for ego assessed between-participant variation in ratings, that is, were some participants consistently providing more positive or negative ratings than others? The alter random effect assessed between-alter variation in ratings, i.e. did some organisations consistently receive more positive or negative ratings from participants? The variance partition coefficient gave an estimate of the proportion of total variation in the outcome at the ego or alter level.

Models also included fixed effects to explore variation in ratings by: (A) study area; (B) Alter characteristics; (C) government/non government organisation; and (D) individual factors such as age and gender. Variables were entered stepwise, beginning with area, alter characteristic, individual characteristics, and finally network characteristics (number of ratings received by alters); this approach describes the overall global variation in ratings, before looking at associations that account for individual and structural influences on positive and negative ratings.

The outcome variable was a 5-point likert rating which was modelled using ordinal logistic regression. The coefficients can be interpreted in a similar way as logistic regression as the odds (or odds ratio) of moving one point up or down the rating scale. This model makes the assumption that all five points are equally spaced, and the association of a variable on moving from one point to the other is equally likely at any point on the scale. When considering perceptions of positive and negative interactions, it’s plausible that the salience of a negative experience may differ from that of a positive one. Brant’s test [11] suggested that the parallel regression assumption held for ego and area level variables, but with more evidence for deviation from the assumption for the alter variables. We report here on the ordinal logistic model which averages the associations across all points of the scale, further detail on variation for more positively and negatively rated services appears in the appendix.

The network data was visualised using ggraph [12], modelling conducted using MCMCglmm [13], and model results visualised using sjPlot [14], all of which are R statistical software packages. The analysis code and an anonymised data extract is available at github.com/Mark-McCann/ICAROS.

#### Qualitative data

Fieldwork notes with demonstrative quotes from each interview were typed up and a thematic analysis [15] applied to the notes by SS and KMcL. The exhaustive coding of all recorded interview quotes were then grouped into higher level themes and sub-themes. Representative quotes that were deemed most representative of each theme were selected for inclusion, the subthemes were presented in full in reports to the local areas during the pandemic.

The qualitative notes and quantitative data were analysed independently. In other words, qualitative themes regarding variability or consistency in positive or negative experiences were written up without reference to the mean ratings for those alter types. MMcC integrated the qualitative and quantitative findings, selecting representative quotes and subthemes from the full report that aligned (whether agreeing or not) with the themes in the quantitative findings i.e. egos and alter types with consistently higher or lower than average ratings.

## Results

Table 1 provides a description of the participants in the study. There were 82 participants in total, we present a complete case analysis based on the 57 (72%) of participants without missing data. The interpretation of the models did not change including those with missing data, but ego level VPC was slightly higher (see appendix for details). Of the 57 participants, 24 (42%) were women, and 23 (40%) rated their health as not good or poor.

**Table 1:** Characteristics of the study participants

	Number of participants (%)
**Areas**	
Area 1	11 (19)
Area 2	5 (9)
Area 3	23 (40)
Area 4	8 (14)
Area 5	10 (18)
**Ego** **characteristics**	Median **(Lower, Upper quartile)**
Age	43 (37,49)
Physical Health	2,84 (2, 3)
Gender	N (%)
Men	33 (58)
Women	24 (42)
Total	57

The participants reported a total of 369 alters with whom they interacted (see Table 2). The most common alter type (22.5%) was interactions with friends and family, followed by addiction services (15.7%), food shopping or food banks (13.3%), and leisure activities (11.7%).

**Table 2:** Alter types, number of ratings and scores for worsening or improvement in alter interactions since COVID19 lockdown

			Rating score			
Alter type	*N* unique alters	Number of ratings	Mean	Centile 25	Median	Centile 75
Pharmacy	41	41	3.83	3	4	5
General Health	23	23	2.48	1	2	3
Food	49	49	2.73	2	3	3
Family/Friends	83	83	2.71	2	3	3
Leisure activities	43	43	2.72	2	3	3
Substance supplier	19	19	3.05	2	3	4
IEP	5	5	3.20	2	4	4
Addiction	23	58	3.07	2	3	4
Peer Support	9	16	3.69	2.75	4	5
Mental Health	22	25	2.48	2	2	3
Social Work	7	7	2.71	2	3	3
**All alters**	324	369	2.93	2	3	4

Rating range from 1 (worse) to 5 (better)

There were also ten alters representing organisations who received ratings from more than one participant; receiving between two and 12 ratings (see Table 3). The mean ratings for alters that received more than one rating were slightly more positive (mean 3.13) than for the full alter dataset (2.93).

**Table 3:** Overlapping alter types, the number of unique services, ratings and average ratings

			Rating score			
Type of alters	*N* unique alters	Number of ratings (Min-Max)	Mean	Median	Centile 25	Centile 75
Addiction	6	41 (3–12)	2.95	3	2	4
Mental Health	1	4 (4–4)	2.75	2.5	1.75	3.5
Peer Support	3	10 (2–6)	4	5	3	5
All alters	10	55 (2–12)	3.13	3	2	5

**Fig. 1:**
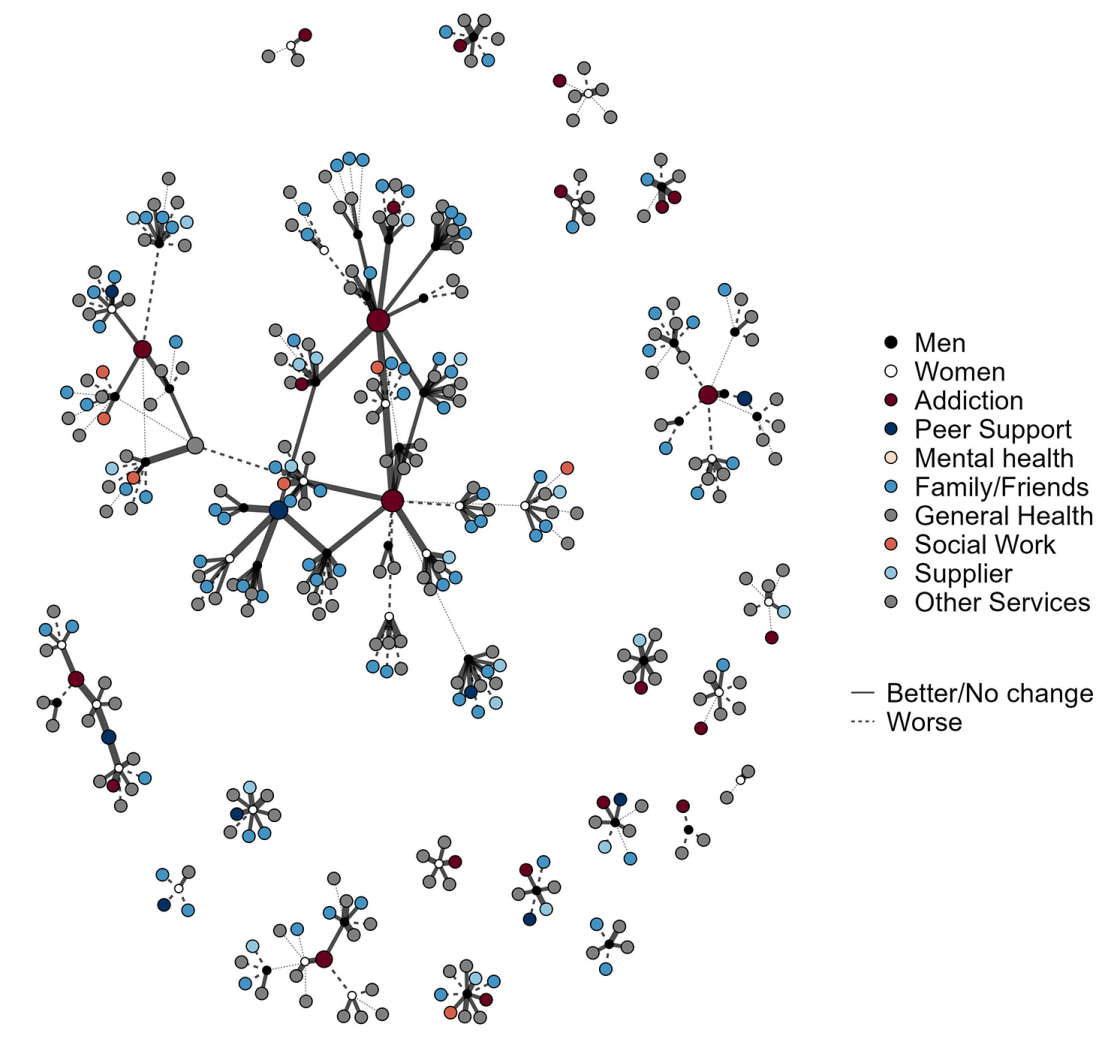
Graph visualisation of participants (black and white), their formal and informal interactions (colours), and interaction rating (wider lines are more positive)

Figure 1 shows a visual representation of the dataset used for analysis as nodes (participant egos or their interaction alters) and ties (the reported rating with those alters). The black (men) and white (women) nodes represent participants, while alters are coloured according to alter type. The node size relates to the number of ratings, with the larger nodes showing the alters receiving multiple ratings, predominantly addiction services and peer support organisations. The lines show a relationship between the participants and their alters, along with positive (wider and solid lines) or negative (thinner and dashed) ratings of that relationship. The bottom right of the figure shows many disconnected egonets, representing individual participants and the number and variety of alters they reported interacting with. To the top left of the figure, there is a group of nodes from a woman reporting only three contacts who reported a negative interaction with one of the three. The centre of the figure shows a group of two addiction services and a peer support service all of which have multiple participants in common. Elsewhere in the figure there are further subcomponents showing services with multiple participants in common. Closer inspection of the figure to identify the light blue alters shows that interactions with non-prescribed substance use suppliers was common, and inspection of the dark red alters shows that almost all of the participants were currently in contact with an addiction service.

This visualisation helps to represent the complex structure of multiple service utilisation, the variety of positive and negative experiences, and how some individuals have a very limited set of formal and informal social interactions. It is worth noting that many of the disconnected components relating to individuals may not be truly disconnected from others in the dataset, there may have been common services, but we were unable to link these in the data collected. While the visualisation gives an overall impression of structure and some potential variation in positive and negative ratings, statistical models give a more useful summary of the complex patterns and variations.

Table 4 shows models of how relationship ratings varied according to fixed effect characteristics of individuals, alters and area, as well as variance partition coefficients (VPCs) describing rating variation between participants and alters. Model 1 includes a random effect for between participant variation in ratings, while model 2 allows for between-participant and between-alter variation in ratings. The VPC for Model 1 suggests that around 10% of the variation in rating relates to between participant differences. That is, some participants were consistently reporting more negative changes to due COVID than the sample average, while others gave more positive ratings. The alter VPC estimate for Model 2 suggests there was around 1% between-alter variation in ratings, meaning that – for the 10 services receiving multiple ratings - there were no services that were consistently receiving better or worse ratings than average. The negligible variation was confirmed by comparing the model fit statistics, which showed no improvement comparing Model 1 (ego variation only) and 2 (ego and alter variation). The conceptual implication is that the system structure is optimally described when accounting for individual differences among those interacting with services, but organisational variation was less variable at the level of individual organisations. Variation between types of organisation was explored in the subsequent models, retaining the random effect terms. Models 3 to 7 show results after the stepwise inclusion of fixed effects for area, ego and alter characteristics. The coefficients for area differences in rating changed markedly after the inclusion of the terms for the type of alters, while there was less change in the association between alter type and rating after the inclusion of ego level characteristics.

**Table 4:** Ordinal logistic multilevel models odds ratio for more positive interaction rating, with participant and overlapping alters cross classified at level two

	1	2	3	4	5	6	7
**Random effects**							
Ego level variance	**1.65 (1.22, 2.39)**	**1.65 (1.19, 2.36)**	1.34 (1, 1.75)	**1.6 (1.16, 2.44)**	**1.63 (1.17, 2.36)**	**1.38 (1.03, 1.9)**	1.38 (1, 1.82)
Alter level variance		1.06 (1.00, 1.23)	1.06 (1.00, 1.26)	1.05 (1.00, 1.27)	1.05 (1.00, 1.23)	1.07 (1.00, 1.35)	1.06 (1.00, 1.3)
Ego VPC	0.1	0.1	0.06	0.1	0.1	0.07	0.07
Alter VPC	~~	0.01	0.01	0.01	0.01	0.01	0.01
**Fixed effects**							
(Intercept)	6.23 (4.66, 8.58)	6.36 (4.48, 8.5)	4.18 (2.41, 7.10)	8.58 (3.60, 25.03)	8.94 (3.60, 25.53)	2.56 (0.91, 8.41)	3.19 (0.92, 12.18)
Area 1			Reference	Reference	Reference	Reference	Reference
Area 2			0.87 (0.38, 1.95)	1.01 (0.43, 2.94)	1.07 (0.41, 2.97)	1.27 (0.52, 3.06)	1.3 (0.52, 2.97)
Area 3			**2.41 (1.36, 4.44)**	**3.22 (1.54, 6.75)**	**3.29 (1.48, 7.17)**	**2.94 (1.55, 5.75)**	**3 (1.48, 5.7)**
Area 4			0.71 (0.36, 1.63)	0.9 (0.29, 2.59)	0.89 (0.3, 2.8)	0.91 (0.33, 2.41)	0.92 (0.35, 2.34)
Area 5			**2.16 (1.04, 4.39)**	**2.77 (1.23, 6.49)**	**2.8 (1.2, 6.49)**	**2.64 (1.19, 5.53)**	**2.59 (1.14, 5.53)**
*Alter characteristics*							
Addiction service				Reference	Reference	Reference	Reference
Leisure activities				**0.54 (0.29, 0.99)**	0.55 (0.29, 1.01)	0.53 (0.30, 1.05)	**0.45 (0.20, 0.93)**
Family Friends				**0.52 (0.29, 0.87)**	**0.53 (0.3, 0.88)**	**0.52 (0.3, 0.91)**	**0.43 (0.23, 0.9)**
Food				0.58 (0.33, 1.13)	0.58 (0.33, 1.11)	0.58 (0.32, 1.09)	0.48 (0.24, 1.02)
General Health				0.48 (0.23, 1.08)	0.48 (0.21, 0.96)	0.51 (0.24, 1.13)	0.43 (0.18, 1.05)
Injecting equipment				2.01 (0.48, 9.58)	2.05 (0.5, 10.28)	2.05 (0.45, 7.69)	1.67 (0.39, 7.1)
Mental Health				0.45 (0.23, 1.00)	**0.44 (0.21, 0.93)**	**0.45 (0.22, 0.97)**	**0.39 (0.17, 0.9)**
Peer Support				2.29 (0.89, 5.42)	2.25 (0.83, 5.53)	**2.32 (1.01, 6.75)**	2.12 (0.76, 5.16)
Pharmacy				**3.63 (1.82, 6.55)**	**3.71 (2.03, 7.1)**	**3.63 (2.03, 6.75)**	**3.03 (1.36, 5.93)**
Social Work				0.76 (0.2, 2.36)	0.76 (0.19, 2.41)	0.82 (0.25, 2.8)	0.68 (0.20, 2.83)
Substance supplier				0.98 (0.44, 2.27)	1.01 (0.44, 2.2)	0.95 (0.44, 2.44)	0.81 (0.31, 1.95)
Non Government Organisation				0.66 (0.34, 1.2)	0.67 (0.35, 1.31)	0.70 (0.39, 1.19)	0.68 (0.39, 1.19)
Alter indegree							0.97 (0.90, 1.04)
*Individual characteristics*							
Gender (Woman)					0.90 (0.57, 1.55)	1.00 (0.64, 1.58)	1.01 (0.62, 1.65)
Physical health						**1.52 (1.22, 1.93)**	**1.54 (1.22, 1.95)**
Deviation Information Criterion	1034.3524	1015.2144	1022.6082	971.349	947.8242	967.247	967.2045

**Fig. 2:**
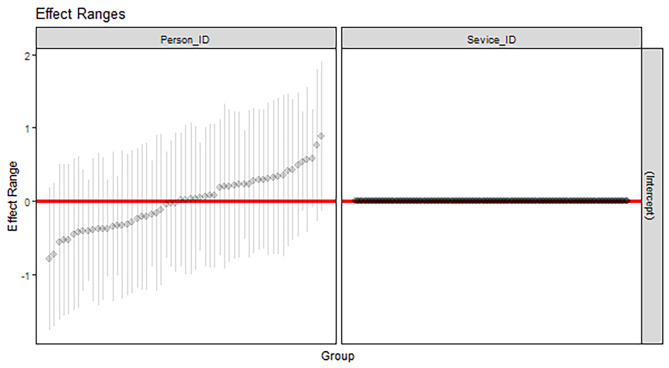
Random effects plots show the distribution of rating scores per ego (participant) and per alter (treatment services receiving multiple ratings). Note: Left panel shows the ego-level variance partition coefficient of 0.07. Right panel shows the alter-level VPC of 0.01

Figure 2 gives a visual representation of the between participant, and between alter variation in ratings. Each vertical line represents one participant, with the position of the dot showing that individual’s average rating and the line showing the variation in ratings. Model 7 found around 7% of the rating variation could be explained as between participant ratings. This can be seen visually in the left panel, with some individuals having ratings higher or lower than the sample average. Looking at the right hand panel, there was virtually no between alter variation with no single service having a much higher or lower than average rating. This was also reflected in the VPC of 1%.

**Fig. 3:**
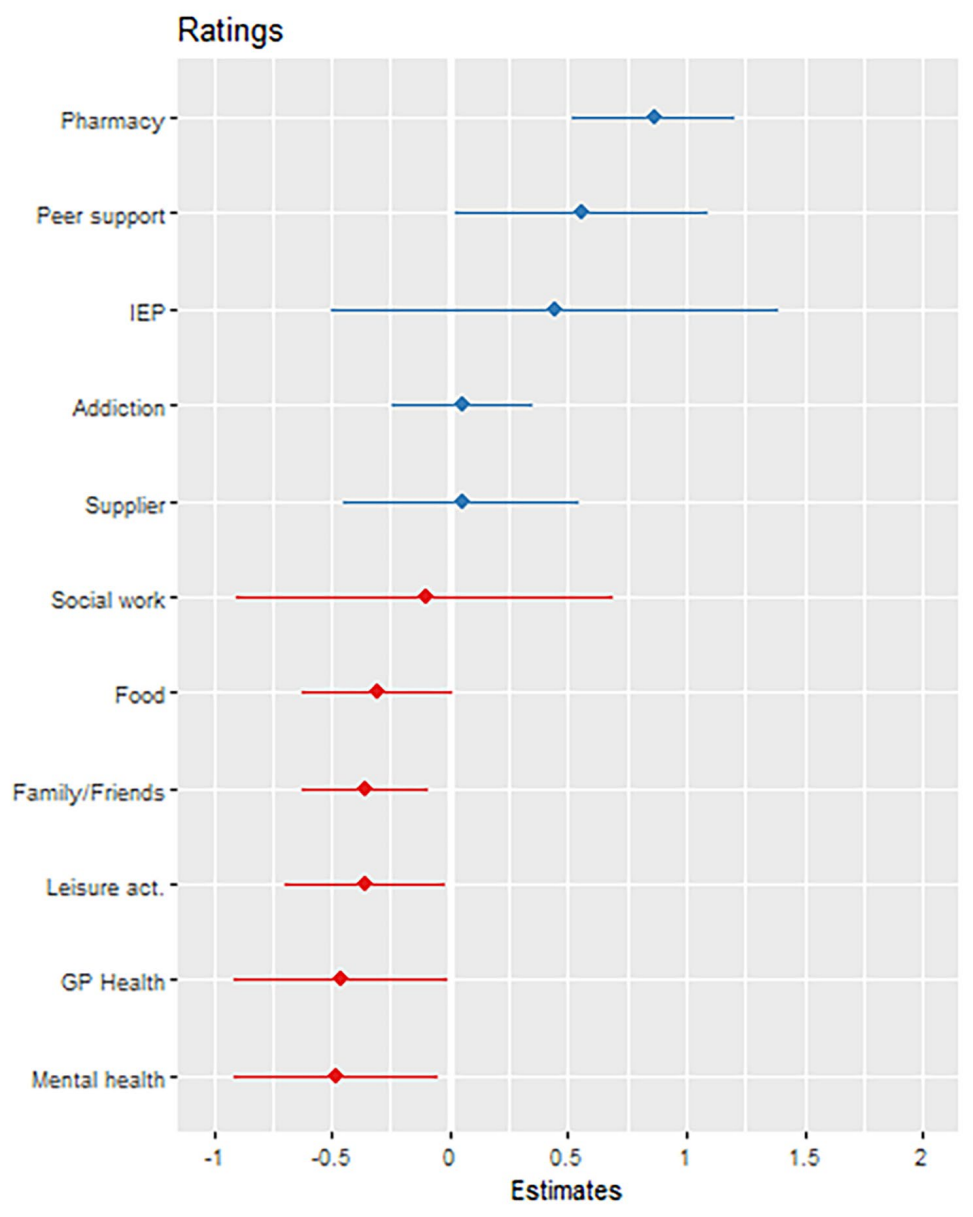
Estimates from an unadjusted linear cross classified multilevel model showing mean (95% Confidence Intervals) for five point rating by alter type. Higher = more positive change in interaction since COVID19.

In terms of participant characteristics, there was no evidence that women reported more or less positively than men, but those who reported better general health tended to provide more positive ratings than those in poorer health (1.52, 95% CI 1.22, 1.93). There were more substantial variations according to alter type. Model 4 shows that the ratings of COVID-related change for pharmacies were most positive, and the 95% confidence intervals suggest that the ratings hadwere consistently more positive than the average rating among the samplehigher odds ratio (Ordinal logistic log odds odds ratio 3.63, 95% CI 1.82, 6.55, ). At the other end of the scale, mental health services were rated more negatively since COVID than average (models 5 to 7, odds ratio in model 5: 0.44 95% CI 0.21, 0.93, ), along with interactions not related to treatment such as family (model 4: 0.52, 95% CI 0.29, 0.87, ), and leisure activities (0.54, 95% CI 0.29, 0.99, ). Pharmacies had more than three times (3.03 95% CI 1.36, 5.93) the odds of getting a 1-point better rating than Addiction services, while there was a lower odds of a more positive rating than an Addiction service for Mental health ( OR 0.39 95% CI 0.17, 0.90), and family/friends ( OR 0.43 95% CI 0.23, 0.90) Fig. 3 gives a visual depiction of the variation in ratings by alter type. Note that the visual is based on a linear unadjusted model rather than the main analytical model in Table 4.

There were notable differences between the areas surveyed, with area 3 being three times as likely to receive a 1-point better rating than Area 1 (OR 3.00 95% CI 1.48, 5.70) after accounting for differences by participant and alter characteristics.

### Qualitative findings

All participants reported that services had changed during COVID19 and in line with restrictions. This meant most support happened over the phone or online and there were mixed views and experiences across all five areas. Some participants said they were talking to workers more often than before COVID19 and found it easy to maintain this. However, many participants reported less contact.

*“Since March 2020, I have had one appointment with a worker at the service. Apart from this there has been no support and very little communication. My script is dropped off at the pharmacy and that’s it. There’s no drug testing and only phone appointments available. I prefer face-to-face contact and struggling with lack of this support.”* (Area 3).

*“I am meant to still speak to them once a month, but it’s been very hard to get in touch with them since Covid. I have struggled to get through.”* (Area 2).

Many participants reported not hearing back from workers when they had tried to reach out for help. There were concerns about missing phone calls from workers, and the impact of this on their ongoing care. Others reported not being able to afford phone credit to initiate contact with services and having to wait a call. Overall, the lack of contact and change in delivery had a negative impact on many of the participants:

*“I’ve not seen my worker during this. It’s terrible as I can’t just go down to the service to see them if needed. My worker is also my trauma counsellor, and we are close. It’s had a bad effect on me.”* (Area 4).

Participants in all areas commented that OST reduction plans had been interrupted or halted, which was often frustrating. There was a general feeling that reasoning and implementation of this were not communicated well:

*“I don’t have a phone at the moment, and I’ve not seen my drug worker in a while. I want to get put down on my script but don’t know how to get in touch with them and I’ve heard no one is getting cut down at the moment.”* (Area 4).

Further demonstrating the varied experiences, some participants felt positively about the remote support offered, with individuals describing how it had increased their ability to engage with support:

*“I’ve enjoyed lockdown and my mental health and emotional health has improved. Normally I’m in the house for long periods of time due to my physical disability and am happy that services are now turning to online platforms. I can access them more and get more support when needed. I hope that things do not go back to face-to-face meetings as I would miss the daily interactions with people. I feel I’m seeing more people now and this is improving my mood.”* (Area 3).

A few participants mentioned they felt it was easier to be dishonest with workers about how they were feeling when support was remote:

*“I think it is easier to lie over the phone and you are less likely to be honest if you aren’t doing okay. You can just say you’re fine even if you’re not. The service was quick to adapt, though, and I have a good worker who links me in with other services.”* (Area 4).

As highlighted here, some participants in each area acknowledged that they felt services had done well to adapt, especially peer support and voluntary services.

### Substance use

Qualitative reports about changes in substance use, quality, availability, and prices varied across the five areas (see Table 5).

**Table 5:** Summary of thematic findings around changes in substance quality and availability after lockdown

Location	Findings summary
Area 1	More methadone available to buy on the street, less cannabis resin and reports of marijuana/grass being laced with “legal highs”.
Area 2	Some participants had relapsed on alcohol; a few were using substances less as not socialising; inconsistencies with prices, quality and cannabis supply.
Area 3	Little change in availability, price and quality of substances. Some differences mentioned around more crack, less heroin and cannabis resin availability, and grass being used more over pollen.
Area 4	Some people had been staying in hostels and tied this to using substances more. Some reports of less heroin and more Valium.
Area 5	Participants reported more substance use among them and their peers. Most reported no change in quality or frequency, one report of less cocaine and cannabis. Multiple reports of Buckfast being more expensive and harder to get.

Relapse or increased use due to impacts of COVID19 was more common in participants overall than a decrease in use:

*“I’ve found the uncertainty of Covid and lockdown hard. This has really affected me mentally and at the start of April I felt extremely low and drank heavily for a week before stopping. I had not drunk alcohol in two years before this.”* (Area 3).

*“I’ve been in oblivion since Covid. I was moved to a hostel which badly affected my social anxiety and there were drugs everywhere. I isolated myself throughout and only went out to the chemist. My drug use increased with heroin and Valium and now I’m in the Crisis Centre. I’m really glad to be here.”* (Area 4).

### Themes for negatively rated alter type: family and friends

Participants in all areas mentioned missing seeing their friends and families due to restrictions limiting contact. Some had not been able to see their children if they did not live with them full time and were finding it difficult to find things to do with them when they did see them.

Some participants described that having children and/or pets helped to motivate them to go outside for walks which could help their well-being:

*“I try to go out for 30 minutes each day, either to a shop or if it is too busy, I just walk around near my house. My daughter has a small dog which I have been walking with a few days a week since the schools went back, which is enjoyable.”* (Area 2).

However, most participants were finding it difficult to pass the time and were overall less active, with impacts on physical health, such as weight gain, described. People mentioned various ways they tried to spend time but, largely, there was a feeling that this was difficult and boredom common:

*“It’s been too cold for walks and the government are telling us to stay at home. I feel numb, every day is the same but I know everyone is going through it. I don’t have internet as couldn’t afford it when I was using it. I mostly just watch telly and am alone with my thoughts so often have flashbacks.”* (Area 4).

### Themes for negatively rated alter type: mental health services

There were inconsistent reports of level of support received from mental health services. Some participants had started engaging successfully with mental health services and there were reports of high levels of daily or frequent support offered to some of those in crisis:

*“Before Covid I was going to counselling once a week. This has been upped by the service to three times a week as I felt like I needed extra support. I am extremely happy with the service and feel extremely supported by them.”* (Area 3).

However, there was more evidence of people struggling to get in touch with mental health services for support, with some feeling they were being passed around and not able to access help they needed:

*“I now have to get a phone appointment and can’t go in to see them face-to-face. I feel they don’t really want to see me to discuss things and just keep giving me more pills”* (Area 2).

*“I’ve had no contact with mental health services since March 2020. I received a letter from the mental health team stating that someone would be in touch. However, I have not heard anything to date. I’m aware that other people I know are getting phone consultations from the same team, but this has not been offered and I’ve found it extremely upsetting. It’s affected my mental health and increased my anxiety.”* (Area 3).

### Themes for positively rated alter type: pharmacies

Almost all of the participants in each area who received Opiate Substitution Therapy (OST) stated that their dispensing arrangements had changed due to COVID-19. For the majority, this meant they were attending the pharmacy to collect their prescription less frequently than before and were able to take it at home between these days. Most participants preferred this new dispensing arrangement:

*“A positive note of the prescribing change was changing from daily dispensing to once per week. I prefer this from having to attend daily and hope this will continue after lockdown.”* (Area 3).

*“I prefer only going once a week. Workers should trust some people more than they do. It’s good for me only going once a week but it won’t be for everyone. It does mean I don’t see people every morning for a chat, though.”* (Area 4).

A few participants did say they prefer going more frequently to the pharmacy and there were mentions of individuals being changed back to going daily/more often. This was for reasons such as relapsing and being pressurised to sell or give their take-home prescription to others. Though in one case, a participant had been returned to frequent pick-ups without any clear communication of why this was happening:

*“I was still on weekly at the start but changed to Monday to Friday pick up again. I think this is because people were selling methadone. I don’t like it like this and don’t know for sure why I’ve been changed from weekly.”* (Area 4).

Participants discussed other new rules that had been adopted by pharmacies due to COVID19, with long queues outside mentioned in all areas. A few individuals did not mind this as felt everyone was being made to queue the same way regardless of what they were there for and thought it showed the pharmacist was sticking to social distancing guidelines. However, for most participants, the queuing outside was negative as they felt more stigmatised while waiting:

*“I was feeling awkward standing in the queue outside and have experienced people shouting abuse at me while waiting. I feel that this happened because people thought I was there to pick up methadone, when in fact I had been waiting to collect my mental health medication.”* (Area 3).

There were also examples of individuals being given or even made to consume their OST whilst still in the queue, as explained here by someone who had other issues with the pharmacy as well:

*“I had to self-isolate two months ago for two weeks due to possibly having Covid. I don’t have any friends or family who could go to the pharmacy to pick up my methadone prescription and because of this I was struck off. I understand that this is because of [service name] but I feel that the pharmacy could have offered to bring my prescription to me. There has been no support from the pharmacy if you are unable to collect your prescription in person. The pharmacy is also making people take their methadone outside on the street. They are asking people to drink it in front of the pharmacy staff. I’m extremely embarrassed by this and would like the respect and privacy that other members of the public have.”* (Area 3).

### Overlap between quantitative and qualitative data

Overall, there was a high degree of overlap between the findings of the thematic analysis and the multilevel modelling. The structure of the interview asked participants to first provide a quantitative rating, and then to provide an explanation and a context for the quantitative rating. The qualitative data provided key insights into the mechanisms underpinning the positive and negative ratings, while the statistical analysis provided greater certainty around the overall trends in ratings. For example, there were frequent thematic pros and cons given in relation to change in pharmacy procedures during COVID by each participant, but the MLM suggests that changes were received positively overall, and the mechanisms through which the positive changes took place.

### Reflection on the project framework

The ICAROS project was initially designed to align with the principles of a Transdisciplinary Complex Adaptive System (T-CAS) approach to health improvement [9]. In practice, it was not possible to achieve this fully. Without large scale funding, it wasn’t possible to capture data at multiple time points in each area, so we provided a single round of feedback to each area rather than initiating learning cycles. At the strategic level, engagement with the findings from the rapid reports varied, with ongoing engagement in some areas, and the report being noted for information in others. In a time and resource-constrained environment, the capacity to develop new networks to support a T-CAS learning cycle was limited. At the community level, the lived experience and academic co-production activity around designing the study provided an effective toolkit for both data collection and practical assessment. For example, data collection identified an individual with a very negative rating for injecting equipment provision, which was explained by the lockdown removing the individual’s peer supply of IEP. The peer team were able to signpost the participant to nearby services at the end of the interview. T-CAS provided a useful guiding framework for considering action and information sharing at multiple levels of the system, and while the project did not initiate positive system disruption and sustained learning cycles, it did provide positive mitigating actions against the wider disruption introduced by the pandemic and lockdown.

## Discussion

This paper reports on a unique peer research led mixed methods social network study design to study system-wide change due to COVID19 within the substance use treatment sector. The findings of the study provided rapid feedback to health professionals across Scotland, and the summative findings presented here demonstrate the value of taking a systems approach to understand complex patterns of change among populations with extensive interactions with a diverse range of health and social care services.

Around 10% of the variation in COVID19 related disruption ratings was due to between-individual differences, this suggests that there were some individuals who were more negatively - and more positively – affected by the pandemic and lockdown than others across all aspects of the informal and formal service interactions. This was explained in part by self-reported health of some individuals, and in part may relate to the extent to which they had a higher need for interaction with services and thus experienced greater disruption. It could be argued that this variation may be due to some individuals having a tendency towards a more positive or negative outlook, rather than an objective change in their interactions across multiple domains. On the one hand, the multilevel modelling approach adequately accounts for the between-individual variation, providing an unbiased assessment of individual, service, and area effects overall. On the other hand, feelings of loneliness and disrupted communications are experiential rather than objective. Our approach identifies those with higher levels of self-reported need. The ability to study both system-wide average trends while also taking a person-centred approach is a key strength of the modelling framework.

A key insight from this study was the extent of variation between types of interaction. Informal interactions with family, friends and social activities were negatively affected. This is fully in line with expectations, as the whole UK population experienced an interruption in their usual patterns of social interactions [1, 16]. For several of the study participants, the rapid change in social support was associated with relapse or an increase in substance use, highlighting the importance relational influences on behaviour. The increase in anxieties and stress due to the pandemic, reduction in social contact, and the variable changes in access to formal services produced disruption to internal resources, social and community resources [5] simultaneously. While this provides lessons on the importance of system preparedness for future disruptive events like a pandemic, is also reinforces the potential for positive intervention at multiple levels to offset negative influences on health compared to e.g. a focus on improving individual resources without supporting access to social and community resources.

This social network study design additionally describes the extent of disruption across the wider health, social care and non-government sector related to substance use. The findings in relation to positive and negative changes mirror what is known from the service provider perspective. Pharmacies experienced rapid and innovative change in procedures [17]. Our findings suggest that changes were in general received positively and outline the processes that explain how a change in delivery led to improved, or worse experiences. A key task for the sector is to identify how to maintain the aspects of system change which produced positive changes, or to find non COVID-related system disruptions which produce positive outcomes.

The study has several limitations. Firstly, the study was designed, and data collected at short notice, with limited resources, and based on a need for rapid information rather than grounded in scientific literature or hypothesis testing. The lack of research grant funding meant that we were unable to collect a larger representative sample or longitudinal data, and instead focussed on cross sectional data in the areas where local funds were available to fund the peer research team. We were only able to construct networks for a subset of the overlapping alters, namely the addiction treatment services and peer support groups where the name of these organisations was recorded during data collection. It is possible that some of the participants had the same GP, Pharmacist, supplier, or friends, but we did not collect detailed name information from every possible alter type. We did this to keep the interview length manageable and reduce participant privacy concerns. Collecting this information may have uncovered a greater level of between-alter variation. For example, it may be that the positive and negative ratings and themes reported for pharmacies related to specific pharmacies. Future service evaluations could consider extending this data collection framework with more detailed information on relevant services and organisations.

Despite some limitations, the study provides useful implications for future research and evaluation. Firstly, we have demonstrated that it is feasible to deploy social network methods to give a system-wide overview of the experience of individuals and the diverse range of interactions taking place within the system. The social network design provides an interview that is adaptive to the individual, so that people in contact with many services, and those not in contact with any services could both give an insight into their current health and social needs and in what ways their needs were being supported or going unmet.

In a recent Delphi study to identify performance measures for addiction treatment services, service users placed a higher priority (than funders or providers) on the extent to which service users are linked up to other services [18]. In a context where services compete against each other for funding, there is a disincentive to make one service’s performance metric dependent on a third party. From a systems viewpoint, and that of individual service users, being able to assess the added value of service coordination and a combination of providers on individual well-being is essential.

The mixed methods approach was able to uncover the diversity of experiences, evidence of trends, and detail on the mechanisms underpinning positive and negative changes related to COVID19. While this study design was created out of the need to attempt to capture the anticipated system-wide effects of the pandemic, the design and the findings suggest that similar approaches could be applied to understand diverse interactions among organisations and people living in the communities in any context. Future research could draw up systems science and network analytic methods to evaluate not only experience of service change, but how formal and informal interactions influence individual health and wellbeing.

### Electronic supplementary material

Below is the link to the electronic supplementary material.

Supplementary Material 1

## Data Availability

The dataset and analysis code supporting the conclusions of this article is available on Github, and on OSF (DOI: 10.17605/OSF.IO/FPB6U, https://osf.io/fpb6u).

## Abbreviations

VPC: Variance Partition Coefficient
COVID19: Coronavirus disease 2019
IEP: Injecting equipment provision
