# Supplementary material for: A social network analysis approach to assess COVID19-related disruption to substance use treatment and informal social interactions among people who use drugs in Scotland
Source: Addict Sci Clin Pract. 2024 May 22;19:42. doi: 10.1186/s13722-024-00469-3 (PMC11110318; doi:10.1186/s13722-024-00469-3)
Supplement: Supplementary file 1 — Supplementary Material 1 [file 13722_2024_469_MOESM1_ESM.docx]

**A social network analysis approach to studying whole system disruption related to COVID19 among people who use drugs in Scotland: Supplementary appendix**

**Appendix A: Consent form**

**
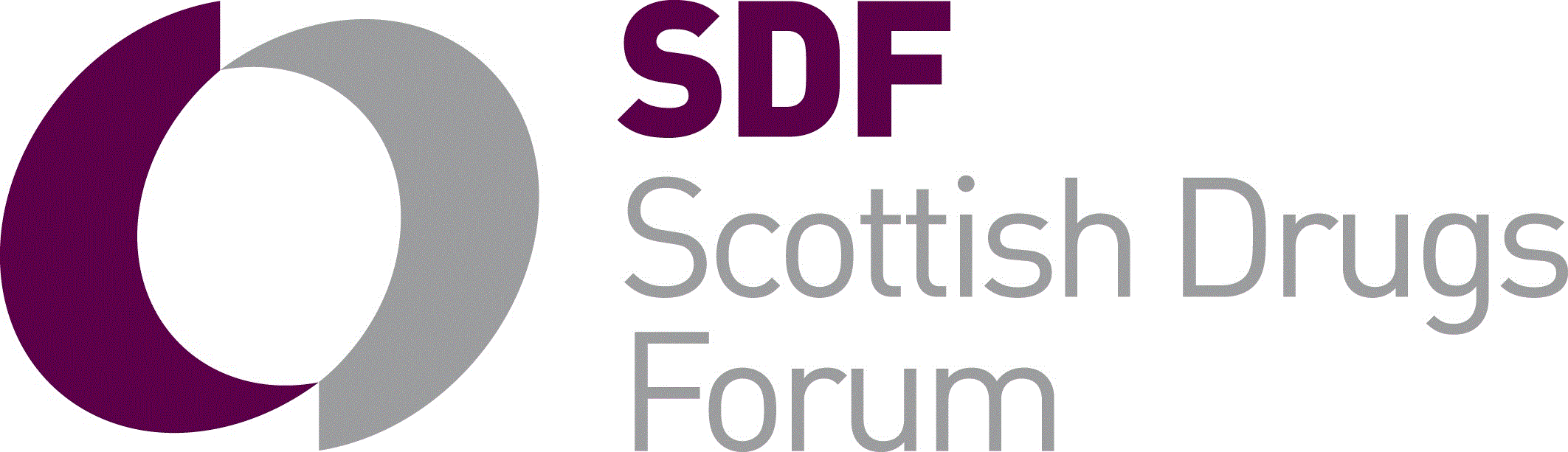
 ICAROS: The Impact of Covid19 and Associated Responses**

**On people who use or have used Substances**

Telephone interview

Consent recording form

This sheet is for SDF development officers to complete notes and collect data from the interview. The peer researchers will read the questions and prompt for follow up information.

- Record notes in the free text boxes about the interview
- Complete the information in all of the tables
  - Use information in the text boxes to fill in the details on the table
  - Complete all boxes, even if to say ‘no change’ or if they don’t use that service
- After completing the form, upload the relevant information to the database [link]
  - Only include anonymous data in the database

**Please record details confirming capacity and consent to interview below, or record notes around postponement or cancellation of interview**

**Notes: _________________________________ Recording officer name: _______________**

**Date: __________________________**

**Participant ID number: ____________________________________**

**Appendix B: Copy of the data collection form used during the peer interviews.**

Telephone interview data collection form

**Participant ID number: ____________________________________**

Thank you for agreeing to take part today. Please feel free to ask me to repeat a question or ask me to explain it if you don’t understand what I mean.

1. **How would you describe your gender? Male/ Female/ Other**
2. **What age are you?**

**Living arrangements**

1. **Where are you living at the moment?**

**Type of accommodation:**

*Own tenancy?*

*Supported accommodation? Single or shared room?*

*With family in their house?*

*With friends in their house?*

*Temporary homeless accommodation? Single or shared room?*

1. **Number of co-residents _________________________**

**Service use**

1. **What services do you regularly use?**

***Only ask supplementary questions for the services they use**

**Service use summary table – add details below**

| **Name of service** | **Use this service** | **Reasons for use** | **How often did you attend before Covid19?** | **How often do you attend since Covid19** | **Has the service Changed since Covid19?** | **Summary of change from discussion** | **Rating of each change**  **Details below** | **Fieldworker notes**  **(not for use in interview)** |
| --- | --- | --- | --- | --- | --- | --- | --- | --- |
|  | Yes /No | Type in | Number  (per week/ per month) | Number  (per week/ per month) | Yes/No | **These changes can be good or bad**  e.g.. take home methadone more injecting equipment being dispensed? Etc – how are you engaging with your worker just now? If not your worker, who are you getting support from? | 1 Much better  2 Better  3 No difference  4 Worse  5 Much worse  6 Mixed (score for each if possible) | Should the service get a structured observation visit, or update on a potential Covid case? |
| **Addiction services**  **Please indicate** statutory or voluntary sector |  |  |  |  |  |  |  |  |
| **Pharmacy** |  | e.g.. pick up methadone/ buprenorphine/ injecting equipment |  |  |  |  |  |  |
| **Health Service** |  | e.g.. wound care, HIV, HCV |  |  |  |  |  |  |
| **Mental Health service** |  | e.g.. psychosocial support/ prescription |  |  |  |  |  |  |
| **Injecting Equipment Provision** |  | Please indicate what type e.g. pharmacy, voluntary sector, van |  |  |  |  |  |  |
| **Other** |  | Please specify: |  |  |  |  |  |  |

**Service change**

1. **What impact are these changes having on you?**

|  |
| --- |

1. **If you have not observed any changes – do you have any concerns about things that may change in the future as a result of Covid19?**

|  |
| --- |

**Substance use change**

1. **Do you currently use street drugs? Yes/ No**
2. **Do you use alcohol regularly? Yes/ No**

**If no to both, skip to Q16**

1. **List of drugs regularly used: _______________________________________________________**

**Alcohol & other drugs summary table. Fill this in after interview**

| **Substance** | **Rough amount** | **Supply price quality change** | **How using** | **Where using** |
| --- | --- | --- | --- | --- |
|  |  |  |  |  |
| **E.g. Benzos** |  |  |  |  |
| Before |  |  |  |  |
| After |  |  |  |  |
| **Alcohol** |  |  |  |  |
| Before |  |  |  |  |
| After |  |  |  |  |
|  |  |  |  |  |
|  |  |  |  |  |
|  |  |  |  |  |
|  |  |  |  |  |

1. **How has drug use changed since COVID19 / Coronavirus / the virus / the outbreak?**

Prompt for changes “What’s it like now? What was usual before?”

Supply, price, quality

Where you’re buying from (more home delivery? Different places?)

Who you’re buying or using with

Where you’re using

Have you had contact with police/ or noticed more police?

1. **Have you noticed any change in supply? Are you worried about this? What will you do if there is a dry up?**

|  |
| --- |

1. **Have you noticed any changes in availability of alcohol? Are you worried about this? What will you do if there is a dry up?** Supply, price, quality; who you’re buying or drinking with; where you’re drinking

|  |
| --- |

1. **How else have things been since Coronavirus?**
   1. How has your health been? Mental health? Physical Health?
   2. What’s it been like going to the shops or getting food?
   3. Anything changed in meeting up with pals? What about with family?
      1. Prompt: Are you/they doing social distancing?
   4. Have you been going out for exercise or fresh air? Have the things you do to pass the time changed?

|  |
| --- |

**General life summary table – fill this in after interview**

| **Topic** | **Change** | **How often go to place** | **Adaptations/**  **Strategies** | **Fieldworker notes** |
| --- | --- | --- | --- | --- |
|  | 1 Much better  2 Better  3 No difference  4 Worse  5 Much worse  6 Mixed (score for each if possible) | If relevant | If relevant | Should the service/business get an observation visit or a potential Covid case update? |
| **Health** |  |  |  |  |
| Before |  |  |  |  |
| After |  |  |  |  |
| **Shops / Food banks** |  |  |  |  |
| Before |  |  |  |  |
| After |  |  |  |  |
| **Family & Friends** |  |  |  |  |
| Before |  |  |  |  |
| After |  |  |  |  |
| **Sport / walks / keeping active** |  |  |  |  |
| Before |  |  |  |  |
| After |  |  |  |  |
|  |  |  |  |  |
|  |  |  |  |  |

1. **Have you heard anything ‘on the street’ from your area about issues other people with a drug problem or in treatment are having?**

Prompt: Are people worried about coronavirus, has anyone had symptoms

|  |
| --- |

1. **Is there anything else you think would be useful for us to know about drug use and COVID19 in your area?**

|  |
| --- |

**We’re doing this study all over Scotland so just want to take a note of whereabouts you are. We won’t post anything to you**

1. **What is the first half of your postcode? ­­­­­­­­­­­­­­­­­­­­­­­­_______________________________________________________________**
2. **Would you be OK if we called you back in a week or two to check if things have changed and how you’re doing? Yes [ ] No [ ]**
3. **Roughly how many people do you know who might be interested in this study?**

Prompt: How many at recovery groups or who is drinking or using other drugs?

**Write in rough number: _____________________**

**That’s the last question. Thank you very much for taking part in the survey!**

**Debriefing**

**Ask for any feedback on the survey, other things we should ask other participants about.**

**Gauge the need for signposting and safeguarding.**

**Appendix C: Analysis for participants with missing data**

The analysis presented in the paper was based on 57 participants with complete data, out of a total of 82 participants. The 25 participants dropped from the complete case analysis had missing data on age, gender, health or all three. A further 109 observations were dropped because there was missing information on whether or not the alter in question was a non government organisation. Figure C1 shows the graph visualisation for the data including missing information. The overall structure is similar, with the additional egos predominantly added as individual components of the graph, but there were a few additional addiction and peer support services who received ratings from several egos.

**Figure C1: Network visualisation of 82 egos and 527 alters.**


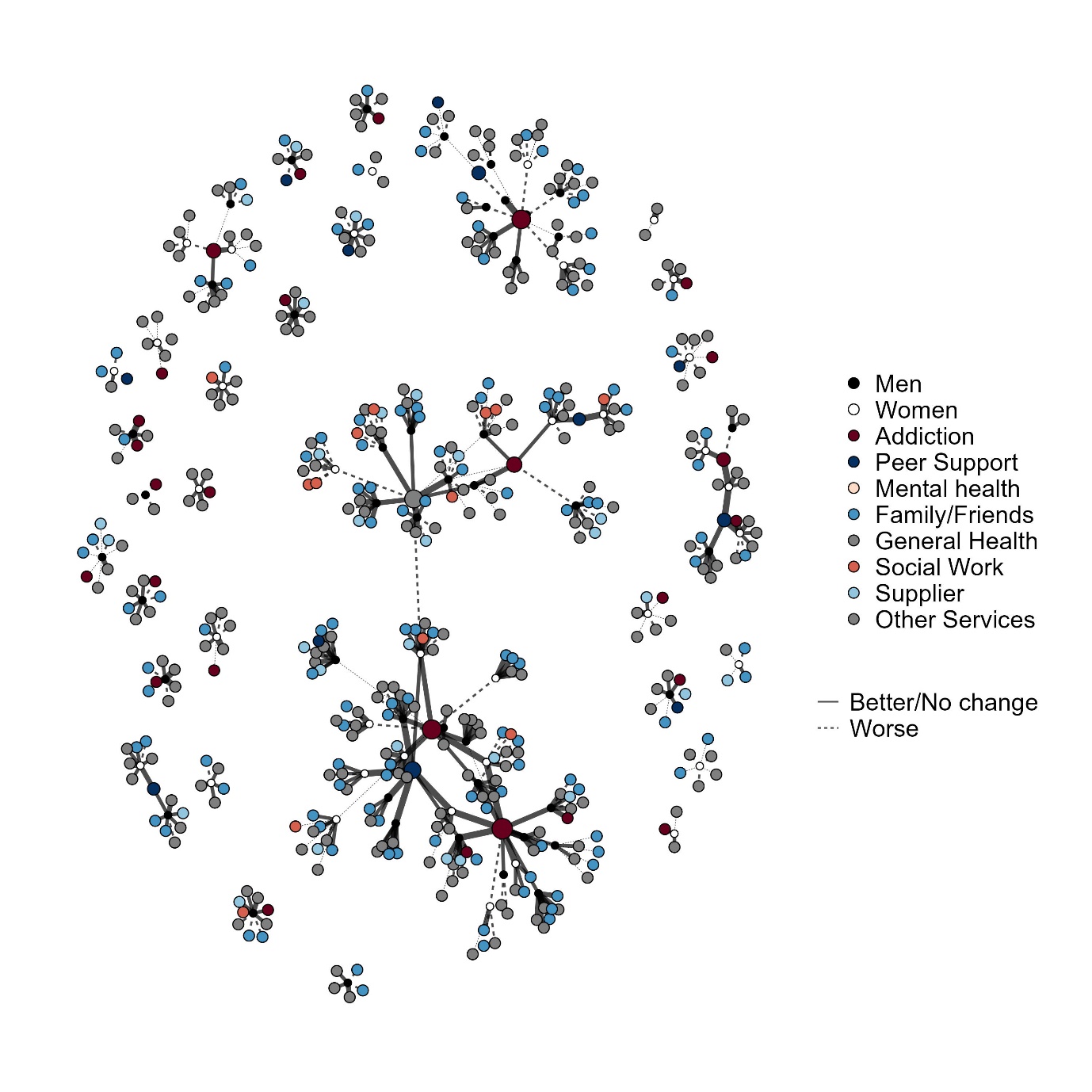


Gender imputed to men for egos with missing data

Table C1 shows the difference between the missing information and complete case datasets according to alter type and mean rating scores. There was little change in the proportions of alter types reported, with a slight increase in the proportion of friend/family contacts (22 to 24%), and a slight drop in the proportion of IEP interactions due to the fact that there were no additional IEP interactions in the extended dataset. The mean rating scores were slightly more negative, suggesting that the complete case analysis may underestimate the extent of negative disruption.

Table C1: Number of alter ratings and mean rating scores for complete case and missing information datasets

|  | **Complete case dataset** | | **Dataset with missing information** | |
| --- | --- | --- | --- | --- |
| **Alter type** | **Number of ratings** | **Mean** | **Number of ratings** | **Mean** |
| Pharmacy | 41 (11.11) | 3.83 | 53 (10.06) | 3.64 |
| General Health | 23 (6.23) | 2.48 | 40 (7.59) | 2.75 |
| Food | 49 (13.28) | 2.73 | 69 (13.09) | 2.75 |
| Family/Friends | 83 (22.49) | 2.71 | 126 (23.91) | 2.63 |
| Leisure activities | 43 (11.65) | 2.72 | 64 (12.14) | 2.66 |
| Substance supplier | 19 (5.15) | 3.05 | 28 (5.31) | 2.79 |
| IEP | 5 (1.36) | 3.20 | 5 (0.95) | 3.20 |
| Addiction | 58 (15.72) | 3.07 | 68 (12.9) | 3.07 |
| Peer Support | 16 (4.34) | 3.69 | 24 (4.55) | 3.54 |
| Mental Health | 25 (6.78) | 2.48 | 37 (7.02) | 2.59 |
| Social Work | 7 (1.9) | 2.71 | 13 (2.47) | 2.77 |
| **All alters** | 369 | 2.93 | 527 | 2.87 |

Table C2 shows the estimates for model 2 (cross classified variance components model) and model 4 (fixed effects for area and alter type). We cannot compare models including ego characteristics due to missing data. The models with missing information showed slightly higher between-ego variation, with a VPC of 14% compared to 10%. The between alter variance changed very little, remaining around 1% of the total variation. There was no substantial change in interpretation of the models, with all coefficients and confidence intervals uncovering the same differences between areas and alter types.


**Table C2: Comparison of complete case and missing information ordinal logistic models**

|  | Dataset with missing information | | Complete case | |
| --- | --- | --- | --- | --- |
|  | 2 | 4 | 2 | 4 |
| **Random effects** | |  |  |  |
| Ego level variance | 1.12 (1, 1.52) | 1.07 (1, 1.34) | 1.06 (1, 1.23) | 1.06 (1, 1.3) |
| Alter level variance | **2.18 (1.48, 3.29)** | **2.1 (1.46, 3.13)** | **1.65 (1.19, 2.36)** | **1.63 (1.15, 2.44)** |
| Ego VPC | 0.15 | 0.14 | 0.1 | 0.1 |
| Alter VPC | 0.02 | 0.01 | 0.01 | 0.01 |
| **Fixed effects** | |  |  |  |
| (Intercept) | 7.24 (5.31, 9.97) | 5.7 (3.13, 11.02) | 6.36 (4.48, 8.5) | 5.81 (2.56, 11.82) |
| Area1 |  | Reference |  | Reference |
| Area 2 |  | 1.9 (0.91, 4.14) |  | 0.95 (0.39, 2.48) |
| Area 3 |  | **3.35 (1.8, 6.23)** |  | **2.83 (1.3, 5.7)** |
| Area 4 |  | 0.8 (0.33, 1.84) |  | 0.59 (0.23, 1.34) |
| Area 5 |  | **2.34 (1.14, 5.05)** |  | **2.36 (1.05, 5.75)** |
| **Alter characteristics** | |  |  |  |
| Addiction service | | Reference |  | Reference |
| Leisure activities |  | **0.51 (0.3, 0.87)** |  | **0.54 (0.3, 0.96)** |
| Family/ Friends |  | **0.48 (0.29, 0.76)** |  | **0.51 (0.29, 0.84)** |
| Food |  | **0.59 (0.34, 0.95)** |  | 0.57 (0.31, 1) |
| General health |  | 0.75 (0.41, 1.45) |  | **0.47 (0.22, 0.94)** |
| Injecting equipment |  | 2.03 (0.51, 9.87) |  | 2.01 (0.47, 8.85) |
| Mental health service |  | **0.52 (0.27, 0.99)** |  | **0.44 (0.19, 0.92)** |
| Peer support |  | 2.08 (0.86, 4.18) |  | 2.23 (0.84, 5.81) |
| Pharmacy |  | **2.72 (1.57, 4.85)** |  | **3.67 (1.99, 6.82)** |
| Social Work |  | 0.73 (0.28, 1.99) |  | 0.76 (0.2, 2.25) |
| Substance supplier |  | 0.66 (0.33, 1.32) |  | 0.99 (0.48, 2.48) |
| Deviation Information Criterion | 1408.1944 | 1348.5446 | 1015.2144 | 968.4655 |

**Table C3: Brant test for the ordinal logistic parallel regression assumption for Model 7**

|  | X2 | df | probability |
| --- | --- | --- | --- |
| Omnibus | 472.823 | 54 | < 0.001 |
| *Individual characteristics* | |  |  |
| Area 2 | 1.904 | 3 | 0.592 |
| Area 3 | 3.512 | 3 | 0.319 |
| Area 4 | 0.062 | 3 | 0.996 |
| Area 5 | 0.639 | 3 | 0.887 |
| Gender (Woman) | 0.887 | 3 | 0.828 |
| *Alter characteristics* |  |  |  |
| Physical health | 4.043 | 3 | 0.257 |
| Leisure activities | 8.418 | 3 | 0.038 |
| Family Friends | 9.149 | 3 | 0.027 |
| Food | 9.564 | 3 | 0.023 |
| General health | 0.455 | 3 | 0.929 |
| Injecting equipment | 5.47 | 3 | 0.14 |
| Mental Health | 7.261 | 3 | 0.064 |
| Peer Support | 0.415 | 3 | 0.937 |
| Pharmacy | 3.49 | 3 | 0.322 |
| Social Work | 13.457 | 3 | 0.004 |
| Substance supplier | 0.739 | 3 | 0.864 |
| Non Government Organisation | 7.672 | 3 | 0.053 |
| Alter indegree | 3.894 | 3 | 0.273 |

Table C3 shows the results for brant’s test of the parallel regression assumption. A low p value for the Chi square test provides evidence that the parallel assumption does not hold. This would mean that the association between a variable in the model and a change in the rating outcome is more pronounced for some values of the rating scale than others. Further analysis using multinomial regression (sample code available on Github), suggests this could be explained by asymmetric responses in the data, for example, peer support services were rarely rated as negative or very negative changes.

Appendix D: STROBE checklist for the project

**STROBE Checklist**

**STROBE Statement—Checklist of items that should be included in reports of *cross-sectional studies***

|  | **Item No** | **Recommendation** |
| --- | --- | --- |
| **Title and abstract** | **1** | **(*a*) Indicate the study’s design with a commonly used term in the title or the abstract** p1 |
|  |  | **(*b*) Provide in the abstract an informative and balanced summary of what was done and what was found** p2 |
| **Introduction** | | |
| **Background/****rationale** | **2** | **Explain the scientific background and rationale for the investigation being reported** p3 |
| **Objectives** | **3** | **State specific objectives, including any prespecified hypotheses** p3 |
| **Methods** | | |
| **Study design** | **4** | **Present key elements of study design early in the paper** p4-5 |
| **Setting** | **5** | **Describe the setting, locations, and relevant dates, including periods of recruitment, exposure, follow-up, and data collection** p4-5 |
| **Participants** | **6** | **(*a*) Give the eligibility criteria, and the sources and methods of selection of participants** p4 |
| **Variables** | **7** | **Clearly define all outcomes, exposures, predictors, potential confounders, and effect modifiers. Give diagnostic criteria, if applicable** p5 |
| **Data sources/** **measurement** | **8******* | **For each variable of interest, give sources of data and details of methods of assessment (measurement). Describe comparability of assessment methods if there is more than one group** p5 |
| **Bias** | **9** | **Describe any efforts to address potential sources of bias** appendix |
| **Study size** | **10** | **Explain how the study size was arrived at** p6 |
| **Quantitative** **variables** | **11** | **Explain how quantitative variables were handled in the analyses. If applicable, describe which groupings were chosen and why** p5 |
| **Statistical** **methods** | **12** | **(*a*) Describe all statistical methods, including those used to control for confounding** p5-6 |
|  |  | **(*b*) Describe any methods used to examine subgroups and interactions** NA |
|  |  | **(*c*) Explain how missing data were addressed** appendix |
|  |  | **(*d*) If applicable, describe analytical methods taking account of sampling strategy** p5 |
|  |  | **(*e*) Describe any sensitivity analyses** appendix |
| **Results** | | |
| **Participants** | **13******* | **(a) Report numbers of individuals at each stage of study—eg numbers potentially eligible, examined for eligibility, confirmed eligible, included in the study, completing follow-up, and analysed** p6 |
|  |  | **(b) Give reasons for non-participation at each stage** p6 |
|  |  | **(c) Consider use of a flow diagram** NA |
| **Descriptive** **data** | **14******* | **(a) Give characteristics of study participants (eg demographic, clinical, social) and information on exposures and potential confounders** p6 |
|  |  | **(b) Indicate number of participants with missing data for each variable of interest** appendix |
| **Outcome data** | **15******* | **Report numbers of outcome events or summary measures** p7 |
| **Main results** | **16** | **(*a*) Give unadjusted estimates and, if applicable, confounder-adjusted estimates and their precision (eg, 95% confidence interval). Make clear which confounders were adjusted for and why they were included** p11 |
|  |  | **(*b*) Report category boundaries when continuous variables were categorized** NA |
|  |  | **(*c*) If relevant, consider translating estimates of relative risk into absolute risk for a meaningful time period** NA |
| **Other analyses** | **17** | **Report other analyses done—eg analyses of subgroups and interactions, and sensitivity analyses** appendix |
| **Discussion** | | |
| **Key results** | **18** | **Summarise key results with reference to study objectives** p19 |
| **Limitations** | **19** | **Discuss limitations of the study, taking into account sources of potential bias or imprecision. Discuss both direction and magnitude of any potential bias** p20 |
| **Interpretation** | **20** | **Give a cautious overall interpretation of results considering objectives, limitations, multiplicity of analyses, results from similar studies, and other relevant evidence** p20 |
| **Generalisability** | **21** | **Discuss the generalisability (external validity) of the study results** p20 |
| **Other information** | | |
| **Funding** | **22** | **Give the source of funding and the role of the funders for the present study and, if applicable, for the original study on which the present article is based** p21 |

***Give information separately for exposed and unexposed groups.**

**Note: An Explanation and Elaboration article discusses each checklist item and gives methodological background and published examples of transparent reporting. The STROBE checklist is best used in conjunction with this article (freely available on the Web sites of PLoS Medicine at http://www.plosmedicine.org/, Annals of Internal Medicine at http://www.annals.org/, and Epidemiology at http://www.epidem.com/). Information on the STROBE Initiative is available at www.strobe-statement.org.**
